# Structural and Chemical Adaptations of *Artemisia monosperma* Delile and *Limbarda crithmoides* (L.) Dumort. in Response to Arid Coastal Environments along the Mediterranean Coast of Egypt

**DOI:** 10.3390/plants10030481

**Published:** 2021-03-04

**Authors:** Ghada A. El-Sherbeny, Mohammed A. Dakhil, Ebrahem M. Eid, Mohamed Abdelaal

**Affiliations:** 1 Department of Botany, Faculty of Science, Mansoura University, Mansoura 35516, Egypt; 2 Botany and Microbiology Department, Faculty of Science, Helwan University, Cairo 11790, Egypt; mohamed_dakhil@science.helwan.edu.eg; 3 Biology Department, College of Science, King Khalid University, Abha 61321, Saudi Arabia; ebrahem.eid@sci.kfs.edu.eg or; 4 Botany Department, Faculty of Science, Kafrelsheikh University, Kafr El-Sheikh 33516, Egypt

**Keywords:** Adaptation, anatomy, drought, essential oils, salinity, phytochemistry

## Abstract

Arid coastal habitats are stressful regions subjected to the effects of biotic and abiotic factors. Vascular plants in these habitats display different responses to cope with these environmental fluctuations. This work addressed the morpho-anatomical features and chemical responses of two medicinal vascular plant species *Artemisia monosperma* Delile and *Limbarda crithmoides* (L.) Dumort., growing naturally along the Mediterranean coast of Egypt. Soil properties (physical and chemical), morpho-anatomical features and chemical constituents (secondary metabolites, antioxidant activity and essential oils) for the two species were performed. Our results displayed that both species are surviving where soils are alkaline, high saline with low moisture and organic carbon. The morphology of both species appeared woody low shrub with fleshy leaves. The most marked anatomical attributes were the thick cuticle of the epidermal layer in leaves and stems, compact palisade cells and abundant idioblasts (secretory ducts, phenolic compounds and calcium oxalate). Also, sclerenchymatous pericycle fibers in stem and glandular trichomes on the leaf had appeared in *A. monosperma*. Both plants exhibited a considerable content of phenolics, flavonoids, tannins, alkaloids and antioxidant activity with a higher level in *A. monosperma* than *L. crithmoides*. The leaf extracts of both plants showed higher values than the stem extracts. The sesquiterpenes group were the major identified compounds of the essential oils (EOs) in *A. monosperma* and *L. crithmoides*, and the majority were oxygenated sesquiterpenes with percentages of 42.63% and 51.49%, respectively. The second major group of EOs was monoterpenes, which were represented in *A. monosperma* in concentrations (34.04%) much higher than those recorded in *L. crithmoides* (4.97%). Exploring the local adaptation mechanism used by the target plants helps us to understand how these plants can acclimatize to harsh conditions, and this provides critical insights into the protection and survival strategy of species under extreme conditions.

## 1. Introduction

Fluctuation in the structure and function of the ecosystem is generally an output of the interactions between its biotic and abiotic components. These interactions are critical in harsh environmental conditions of coastal and arid lands, so any defect in any component of the ecosystem leads to fundamental changes in others, thus establishing a distinct microhabitat [1]. Coastal habitats are depicted by harsh conditions owing to the impacts of biotic and abiotic factors. These conditions stimulate a zonation of plant communities and high ecological diversity along a gradient from the coastal to inland regions [2,3].

Egypt’s Mediterranean coastal land is a part of the dry arid climatic areas and is threatened by natural and anthropogenic factors [4,5]. The most significant abiotic stress agents that affect plant communities in the coastal and arid habitat are climatic-induced processes of erosion and deposition, temperature, drought, salinity, substrate instability and nutrient deficiency [3,6]. Biotic stress is induced not only by human impacts but also by other living organisms such as herbivores, weeds (allelopathy and competition) and parasites [5,7]. Consequently, plant species of these habitats acquired different adaptation or avoidance strategies against stressful abiotic and biotic agents. Such strategies include adaptation like modifications of morphological and anatomical structures, plant chemo-strategies, antioxidant activity, etc. [8,9,10]. Morpho-anatomical characters of different plant organs growing in harsh conditions are deemed significant as acclimatization reflects the ecology of a particular species habitat [11]. Some studies have conveyed a relationship between particular morpho-anatomical features and drought stress resistance in plant stems [11,12]. Other researches revealed that the elevated levels of salinity result in anatomical modification like leaf thickness, reduction in stomatal number and idioblasts’ appearance [12,13]. On the other hand, plant chemo-strategies include the production of different secondary metabolites (SMs) from the primary constituents. These SMs are defined as constituents that have no essential function in the maintenance of life processes, but they participate in defensive roles as a response to biotic and abiotic stresses [14].

Generally, there is little information about tolerance and avoidance mechanisms of psammophytic and halophytic species in arid-coastal regions. In the present study, two Asteraceae species: *Artemisia monosperma* Delile and *Limbarda crithmoides* (L.) Dumort. were selected. The two plant species are among the dominant and poorly-characterized species along the Mediterranean coastal region of Egypt. *Artemisia* is a genus of small herbs and shrubs. This genus includes more than 500 species distributed in North America, Europe and Asia [15]. Many species of the *Artemisia* genus are oil-producing and used for medicinal purposes [16]. *Artemisia monosperma* is a psammophytic shrubby perennial species distributed in the East Mediterranean region and Arabian Peninsula. In Egypt, it grows in desert plains, and inland or coastal wadis within the Mediterranean coastal sector. The medicinal importance of *A. monosperma* is attributed to its anticancer, antimalarial, antispasmodic, antihypertensive and antioxidant activities [17,18,19]. Also, the insecticidal, larvacidal and antimicrobial properties of *A. monosperma* are due to the bioactive compounds and essential oils (EOs) [20,21,22]. On the other hand, *Limbarda crithmoides* (synonymous = *Inula crithmoides* L.) is natively distributed along the wetlands and salt marshes of the Mediterranean Sea and Europe. In Egypt, it is among the common halophytic medicinal species in the deltaic Mediterranean coast. It is frequently consumed by grazing animals. *L. crithmoides* is a perennial halophyte with salt-tolerant features, antioxidant, biological activity, and commonly used in traditional medicine [23,24,25,26,27]. Due to its high iodine and vitamin content, *L. crithmoides* is eaten with vinegar in Lebanon and Spain [23,28].

Globally, salinity and drought reduce the growth and productivity of plants [29,30]. Freshwater scarcity is expected to rise in the future and consequently, the impact of abiotic stresses will increase. Therefore, there is an urgent need to understand the adaptation strategies of plants naturally growing in such stressed habitats. We assumed that both selected species are equipped with a specific adaptation strategy that enables them to survive and persist in these harsh conditions. Thus, this study aimed to assess the morphological, anatomical, and chemical behaviors of *A. monosperma* and *L. crithmoides* in their arid coastal environments along the Mediterranean coast of Egypt.

## 2. Results

### 2.1. Soil Properties

The soil data in Table 1 showed that the soil texture of site I of *A. monosperma* was sandy soil, and site II of *L. crithmoides* was sandy-loamy soil. The soil moisture content varied from 3.50% to 4.70%, where the water-holding capacity ranged from 50.30% to 62.86% at the two sites, respectively. Soil pH shifted to a moderately alkaline range and varied from 7.81 to 8.21 at sites I and II, respectively. Electric conductivity was 6180 µS/cm at the site I and 7730 µS/cm at site II, indicating a high salinity. Bicarbonates, chlorides, and calcium carbonates, which reflect the soil salinity status were higher in site II than the site I. The soils of sites I and II were characterized by a low organic matter content (0.90% and 1.40%, respectively).

### 2.2. Morphological and Anatomical Features of A. monosperma and L. crithmoides

**Figure 1:**
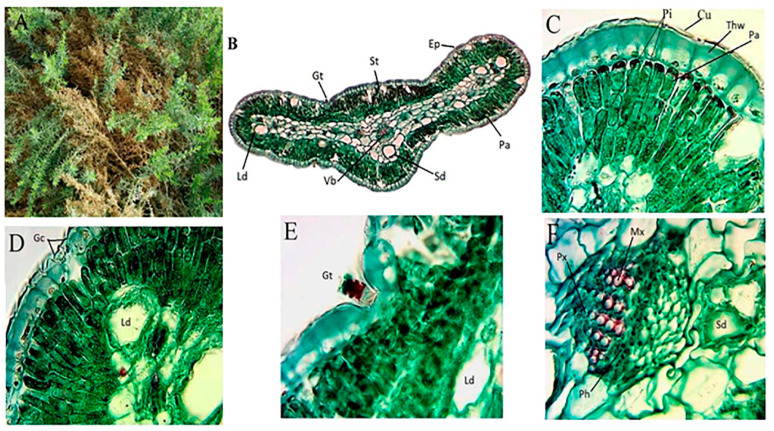
Morphology and anatomy of *A. monosperma* leaf. (**A**) Morphology; (**B**) A transverse section in leaf showing the general view, Ep: epidermis, St: stomata, Pa: palisade tissue, Sd: schizogenous duct, Vb: vascular bundle; (**C**) cuticle (Cu), Thw: thick wall, Pa: palisade tissue, Pi: phenolic idioblasts; (**D**) guard cells (Gc), Ld: lysigenous duct; (**E**) glandular trichomes (Gt) and (**F**) enlarged vascular bundle (Vb), Mx: metaxylem, Px: protoxylem, Ph: phloem. Bar: B = 40 µm, C–F = 400 µm.

**Figure 2:**
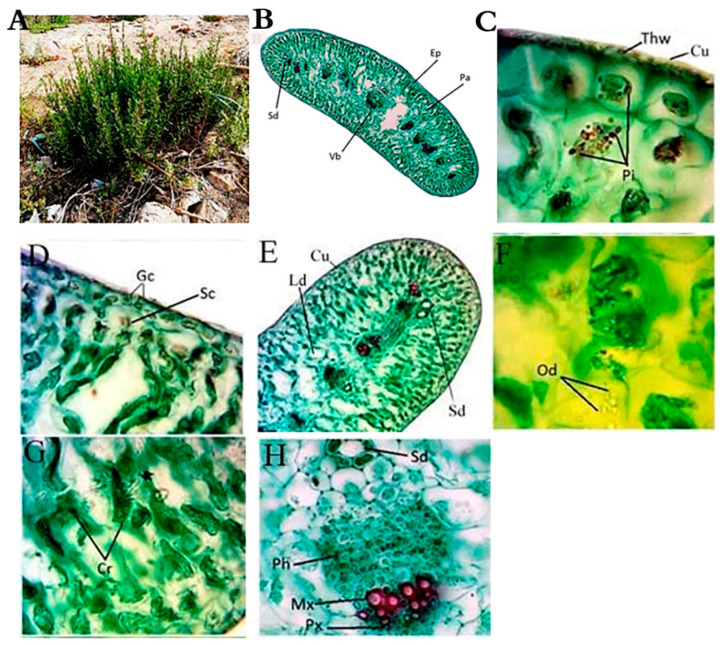
Morphology and anatomy of *L. crithmoides* leaf. (**A**) Morphology, (**B**) A transverse section in leaf showing the general view, Ep: epidermis, Pa: palisade tissue, Sd: schizogenous duct, Vb: vascular bundle; (**C**) phenolic idioblasts (Pi), Thw: thick wall, Cu: cuticle; (**D**) guard cells (Gc), Sc: substomatal chamber; (**E**) lysigenous duct (Ld), Sd: schizogenous duct, Cu: cuticle; (**F**) Oil drops (Od); (**G**) crystals (Cr) and (**H**) enlarged vascular bundle (Vb), Mx: metaxylem, Px: protoxylem, Ph: phloem, Sd: schizogenous duct. Bar: B = 40 µm, C–H = 400 µm, E = 100 µm.

*Artemisia monosperma* is a woody glabrous low shrub with numerous branched stems and pallid green fleshy leaves. Leaves of vegetative branches are oblanceolate in outline, pinnatisect, with linear acute lobes oblong to slightly clasping at the base (Figure 1A). *L. crithmoides* is a woody glabrous low shrub, glabrous, solid, long and cylindrical stem, with lateral monopodial nature of branching and has active floral terminal and vegetative axillary buds. (Figure 2A).

The leaf shape of *A. monosperma* is isobilateral while the shape of *L. crithmoides* is cylindrical bifacial (Figure 1 and Figure 2B). The outer walls of the epidermis were covered by a thick wall and cuticle layer in the two studied plants (Figure 1C and Figure 2C). In both plants, the stomata have small guard cells and a well-defined substomatal chamber, but their abundance was higher on the *A. monosperma* leaf. The mesophyll region consists of elongated and compactly arranged palisade cells in both plants where the central region is occupied by thin-walled living spongy parenchyma in *A. monosperma* and loosely arranged spongy parenchyma with intercellular spaces in *L. crithmoides* (Figure 1D and Figure 2D). Phenolic idioblasts were noticed in the epidermal and subepidermal layers which appeared in a black red color in both plants (Figure 1C and Figure 2C). Also, there are many schizogenous and lysigenous ducts within the spongy tissue in both plants (Figure 1B,D,E,F and Figure 2B,E,H, respectively), but the abundance was higher in *A. monosperma*. The epidermal layer of *A. monosperma* was featured by glandular trichomes which were absent in *L. crithmoides* leaf (Figure 1E). Calcium oxalate crystals and oil drops were recorded in *L. crithmoides* leaf (Figure 2G,F). The vascular bundles were open collateral with hardly distinguishable cambium (Figure 1F and Figure 2H, respectively).

**Figure 3:**
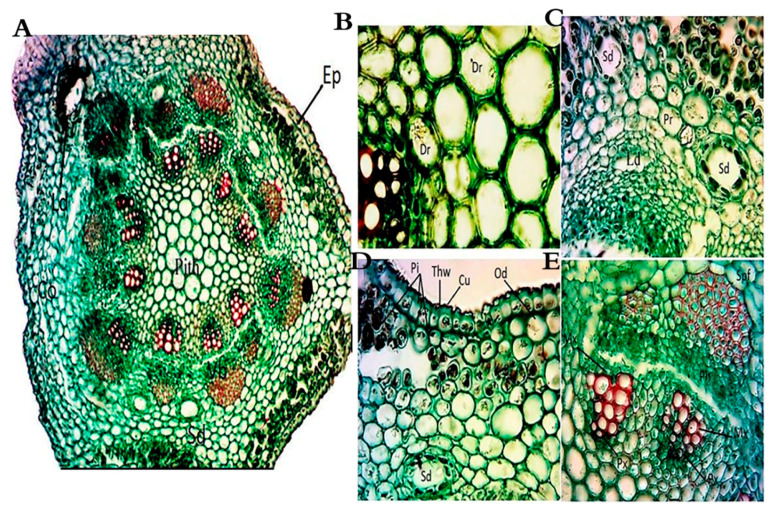
Anatomy of *A. monosperma* stem. (**A**) A transverse section in stem, Ep: epidermis, Co: cortex, Lc: lysigenous duct, Sd: schizogenous duct, Vb: vascular bundle; (**B**) Druses crystals (Dr); (**C**) Prismatic crystals (Pr), Lc: lysigenous duct, Sd: schizogenous duct; (**D**) thick wall (Thw), Cu: cuticle, Pi: phenolic idioblasts, Od: oil drops; (**E**) enlarged vascular bundle (Vb), Mx: metaxylem, Px: protoxylem, Ph: phloem, Spf: sclerenchymatous pericycle fibers. Bar: A = 40 µm, B–F = 400 µm.

**Figure 4:**
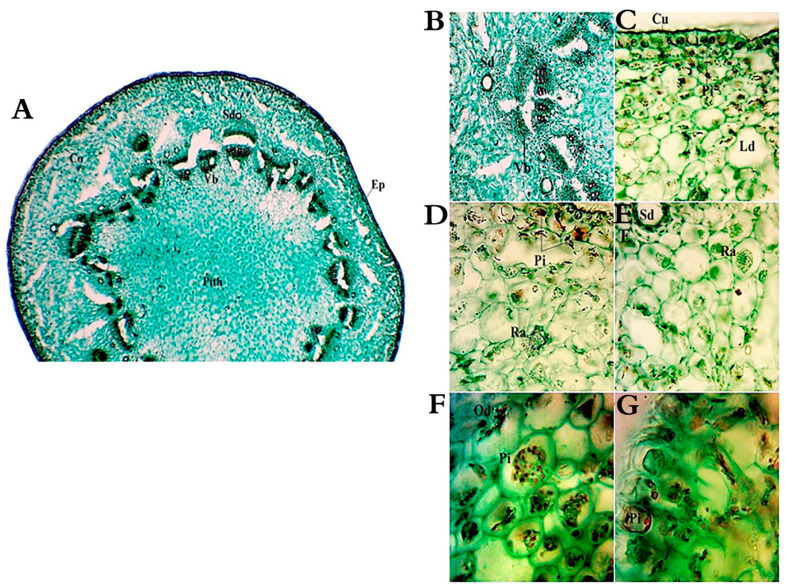
Anatomy of *L. crithmoides* stem. (**A**) A transverse section in stem, Ep: epidermis, Co: cortex, Sd: schizogenous duct, Vb: vascular bundle; (**B**) enlarged vascular-bundle (Vb); (**C**) cuticle (Cu), Pi: phenolic idioblasts, Ld: lysigenous duct; (**D**,**E**) raphides crystals (Ra); (**F**,**G**) oil drops (Od). Bar: A = 40 µm, B–E = 100 µm, F–G = 400 µm.

The cross-sections in the stems of *A. monosperma* and *L. crithmoides* showed typical dicot stems with an epidermis of a single layer of compact barrel-shaped cells without intercellular spaces covered with a thick cuticle (Figure 3A and Figure 4A). The stem shape of *A. monosperma* is wavy, so the cortex consists of the protuberance, which is composed of 5–8 layers of collenchyma, and furrows, which consists of 3–4 layers of chlorenchyma. On the other hand, the stem of *L. crithmoides* is circular in transverse section with the cortical parenchyma zone composed of 4–9 layers of irregular isodiametric parenchymatic cells. Also, the cortex layer is characterised by the presence of schizogenous and lysigenous cavities in *A. monosperma* (Figure 3A,D) whereas *L. crithmoides* is characterised by abundant schizogenous ducts (Figure 4A,B,E). Calcium oxalate of druses and prismatic types were recorded in *A. monosperma* (Figure 3B,C), and abundant raphids were recorded in *L. crithmoides* (Figure 4D,E). Phenolic compounds and oil drops near the outer cortical cell of the stem were observed in *A. monosperma* (Figure 3D) and *L. crithmoides* (Figure 4D,F,G). Well-developed conductive tissues consist of xylem and phloem in open collateral vascular bundles in both plants. Beneath the endodermis, there is a massive zone of sclerenchymatous polygonal pericycle fibers which form a cap-like structure over the bundles in *A. monosperma* (Figure 3E).

### 2.3. Phytochemical Analysis of A. monosperma and L. crithmoides

#### 2.3.1. Secondary Metabolites

**Figure 5:**
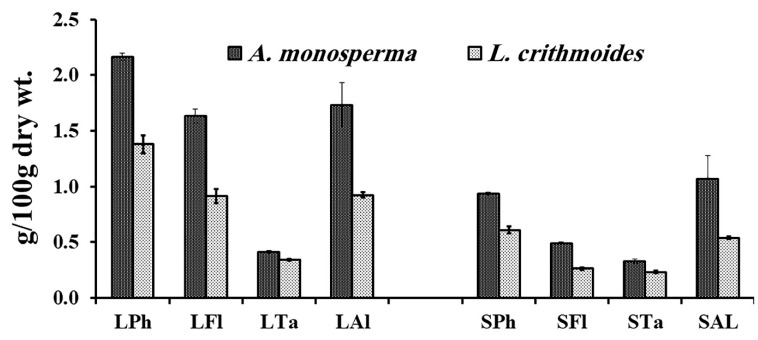
Phytochemical analysis of leaves and stems of *A. monosperma* and *L. crithmoides*. L = leaf, S = stem, Ph = phenols (g GAE/100 g dry wt), Fl = flavonoids (g CE/100 g dry wt.), Ta = tannins (g TAE/100 g dry wt), Al = alkaloids (g/100 g dry wt.). GAE: gallic acid equivalents, CE: catechin equivalents, TAE: tannic acid equivalents.

The results displayed in Figure 5 revealed that both studied plants possess a considerable content of total phenols, flavonoids, tannins and alkaloids with higher content in *A. monosperma* than in *L. crithmoides*. The leaf extracts of both plants exhibited higher values than stem extracts.

The total phenols content in *A. monosperma* leaves (2.17 g gallic acid equivalents (GAE)/100 g dry wt.) was higher than that in *L. crithmoides* leaves (1.38 g GAE/100 g dry wt.) with a significant difference (t = 15.8, *p* < 0.001). Total phenols content in stems significantly differed between *L. crithmoides* (0.93 g GAE/100 g dry wt.) and *A. monosperma* (0.61 g GAE/100 g dry wt.). The flavonoids content in *A. monosperma* leaves (1.63 catechin equivalents (CE)/100 g dry wt.) was significantly (t = 17.8, *p* < 0.0001) higher than that of *L. crithmoides* leaves (0.92 CE/100 g dry wt.). Also, the stem showed the same trend where flavonoid content in *A. monosperma* stem was 0.49 CE/100 g dry wt. and recorded 0.26 CE/100 g dry wt. with a significant difference (t = 34.0, *p* < 0.0001). Tannins concentration in *L. crithmoides* leaves (0.41 g tannic acid equivalents (TAE)/100 g dry wt.) was significantly (t = 7.4, *p* < 0.01) lower than that of *A. monosperma* leaves (0.34 g TAE/100 g dry wt.). Also, the tannins level in the stem ranged from 0.24 g TAE/100 g dry weight in *L. crithmoides* to 0.32 g TAE/100 g dry weight in *A. monosperma* (t = 7.5, *p* < 0.01). The alkaloids content in leaves significantly ranged from the highest value in *A. monosperma* 1.73 g/100 g dry weight to the lowest value in *L. crithmoides* 0.92 g/100 g dry weight (t = 4.6, *p* < 0.05). The stem of both plants exhibited a similar trend with a significant difference (t = 4.4, *p* < 0.01) from the highest alkaloids content in *A. monosperma* stem (0.94 g dry wt.) to the lowest content in *L. crithmoides* stem (0.54 g dry wt.).

#### 2.3.2. Antioxidant Activity 

**Figure 6:**
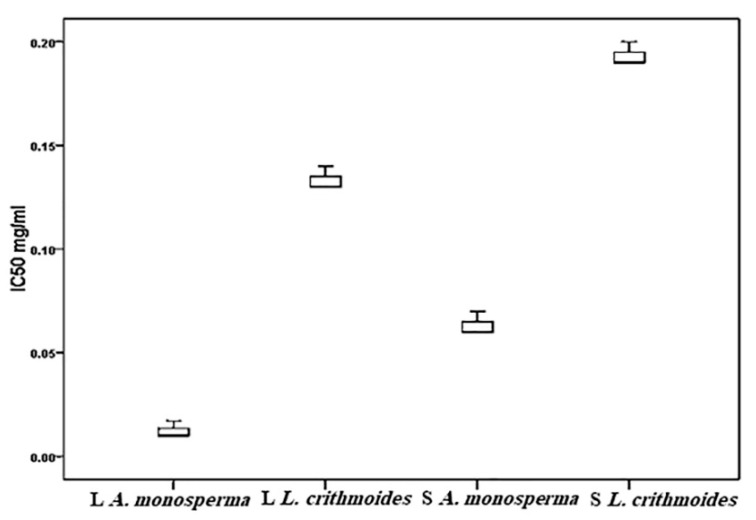
DPPH (1,1-diphenyl-2-picrylhydrazyl) radical scavenging capacity (IC50) in the leaf (L) and stem (S) of *A. monosperma* and *L. crithmoides*. Mean values are significantly different at *p* < 0.05.

The antioxidant activity of *A. monosperma* and *L. crithmoides* was estimated by DPPH (1,1-diphenyl-2-picrylhydrazyl) assay which exhibited elevated antioxidant scavenging activities with values higher or equal to that of ascorbic acid in ethanol extract (Figure 6). The ethanolic extracts of *A. monosperma* and *L. crithmoides* leaves showed significantly different IC50 values, ranging from 0.01 to 0.13 mg/ml, respectively (t = −29.7, *p* < 0.0001). The antioxidant scavenging activity in stems varied from 0.06 mg/ml in *A. monosperma* to 0.19 mg/ml in *L. crithmoides* (t = −27.6, *p* < 0.0001).

#### 2.3.3. Essential Oils Characterization

The chromatograms and structure of the main essential oils (EOs) of *A. monosperma* and *L. crithmoides* are shown in the Supplementary Materials (Figures S1 and S2). The results displayed that EOs of *A. monosperma* and *L. crithmoides* include 30 compounds for each plant (Table 2). Three groups of organic EOs components (monoterpenes, sesquiterpenes and hydrocarbons), in addition to the diterpene group, were recorded in *L. crithmoides*.

The sesquiterpenes group was the major identified compound of the EOs in *A. monosperma* and *L. crithmoides*, which recorded 48.35% and 64.47%, respectively. Grossly, the identified sesquiterpenes were categorized into (1) oxygenated sesquiterpenes with a percentage of 42.63% and 51.49%, respectively and (2) non-oxygenated sesquiterpenes of 5.72% and 12.98%, respectively. The common oxygenated sesquiterpenes components in the studied plants were γ-eudesmol (14.66% and 8.26%, respectively), viridiflorol (5.62% and 8.15%, respectively), citronellyl iso-valerate (2.76% and 4.09% respectively) as well as a considerable content of hexahydrofarnesyl acetone, (−)-spathulenol, guaiol and (−)-caryophyllene oxide. Concerning non-oxygenated sesquiterpenes compounds, α-curcumene (5.72% and 7.49%, respectively) was the common between *A. monosperma* and *L. crithmoides* and the only non-oxygenated sesquiterpenes compound recorded in *A. monosperma*. While, α-gurjunene, α-selinene, beta-caryophyllene, germacrene D were recorded in *L. crithmoides*.

The second major detected group of EOs was monoterpenes, which are represented in *A. monosperma* with a concentration (34.04%) greatly higher than that recorded in *L. crithmoides* (4.97%). Overall, this group includes oxygenated monoterpenes with a percentage of 21.45% and 3.68%, respectively, where (−)-citronellol (3.01% and 1.01, respectively) was the common compound between the studied plants. Other oxygenated monoterpenes in *A. monosperma* were linalool, (−)-*trans*-pinocarveol, pinocarvone, 4-terpineol, (−)-myrtenol, cuminic aldehyde, and bornyl acetate as well as in *L. crithmoides*, benzaldehyde, 4-(1-methylethyl)- and geranyl acetate were detected. Concerning the second group of monoterpenes, monoterpene hydrocarbons of EOs were 12.59% and 1.29% in *A. monosperma* and *L. crithmoides*, respectively. Only *p*-cymene (1.46% and 1.29%, respectively) was the common between the two plants and the only monoterpene hydrocarbon recorded in *L. crithmoides*. Other monoterpene hydrocarbons in *A. monosperma* were (±)-β-pinene (6.35%), D-limonene (2.09%), α-pinene, and γ-terpinene.

The oxygenated diterpene EOs were only detected in *L. crithmoides* and represented only by one compound *cis*-abienol (1.24%). Finally, the hydrocarbons EOs group varied from 17.61% in *A. monosperma* to 29.32% in *L. crithmoides*. The common hydrocarbons between the two plants were 1,1′-biphenyl (6.59% and 11.77% respectively) and naphthalene, 2-ethenyl (5.24% and 8.83%, respectively).

## 3. Discussion

In this study, the coastal soil supporting the studied plants (*A. monosperma* and *L. crithmoides*) was characterized by its alkaline range, sandy to sandy-loamy texture, low moisture content, low water holding capacity, low fertility and high salinity. These results agreed with studies that reported that the Mediterranean regions have different types of soils and featured by a low organic matter content, slightly acidic to alkaline pH and medium to low fertility [3,5]. The deficiency of organic matter (fertility) may be attributed to the low content of micro-organisms in arid soils which lead to a reduction in the soil water-holding capacity [31].

The plants growing in saline habitats have manifested various strategies that help them to cope with these extreme habitats. Such strategies are often exhibited in the morphological, anatomical, and phytochemical alterations of plants [12,32]. Morphologically, *A. monosperma* and *L. crithmoides* plants are featured by their shrubby hard, and woody stems, which aid to resist wind in coastal habitats. The fleshy features of leaves in both studied plants may be due to excessive mucilage to store maximum water, which has a significant role in the dilution of salt concentrations [29].

Also, the leaves of both studied plants were reduced in size which helps them to avoid excessive transpiration. Microscopy investigation revealed that the leaf surface is covered by a thick cuticle and waxy layer to reduce water loss from the leaf surface which is completely justified by the improved tolerance to extreme moisture [11,33]. The leaf shape of *A. monosperma* is isobilateral, which is reported as a prevalent feature of plants growing in arid and hot habitats to decrease leaves’ heating and transpiration requirements [34]. *L. crithmoides* leaf is rather cylindrical which protects the stomata that occur in furrows and prevent direct contact to the atmosphere. In *L. crithmoides*, the stomata were very reduced in number and size per area. The mesophyll region consists of compactly arranged chloroplast containing cells; this compact arrangement helps to check transpiration. Also, the palisade cells remain radially elongated to minimize the direct penetration of light [34].

The stem of *A. monosperma* is featured by its wavy shape and its cortex which is differentiated into collenchyma and chlorenchyma. The main function of collenchyma cells is to strengthen the organ, while the chlorenchyma aids in the photosynthesis process. The epidermal wall of both plant stems is featured by a thick wall, which can reduce water loss and contribute to the plant bearing high temperatures [35]. The stem of *A. monosperma* was characterized by the presence of cap-like sclerenchymatous fibers at the vascular bundle side, which provide hardness to the stem and hence are difficult to chew with the wind. These results agreed with [13,35,36].

There are abundant lysigenous and schizogenous ducts spread within the spongy tissue in the leaves and cortex of the stem in the studied plants. Also, idioblasts containing calcium oxalate crystals are found in the spongy leaf tissue of *L. crithmoides*. In this context, the presence of secretory structures in certain families, such as Asteraceae and Fabaceae, gave more adaptive prosperity in multiple environments [36]. Moreover, the appearance of secretory organs in the same family was reported for Asteraceae, Fabaceae, Salicaceae, Moraceae, Araceae and Cornaceae that are widely distributed in tropical areas [36]. Secreted compounds may be greatly affected (increased or decreased) when the plant is exposed to various environmental stresses, like infections, wounds or edaphic or climatic factors [37].

The plants’ secondary metabolites (SMs) are considered as chemo-strategies for defense reactions to environmental stresses as drought and salinity [38,39]. As a protection against these conditions, oxidative stress was induced as antioxidants. The antioxidants can be classified into two classes, enzyme antioxidants, and non-enzymatic constituents part of the non-enzymatic constituents, the secondary plant metabolites comprising the flavonoids and non-flavonoid polyphenols [40,41].

The results obtained revealed that *A. monosperma* and *L. crithmoides* possess a considerable amount of SMs as phenolics, flavonoids, tannins and alkaloids in extracts of their leaves and stems. Some studies reported that the rise of salinity results in an increase in the content of plant polyphenol as in the alkaloids content of *Achillea fragrantissima* and *Solanum nigrum* [42]. On the other hand, in response to drought, there is a rise in the level of plant secondary metabolites as in *Catharanthus roseus*, *Hypericum brasiliense* and *Artemisia annua* [42]. Moreover, a noticeable variation in the levels of volatile compounds was recorded in *Thymus vulgaris* under water-stress conditions [43].

Volatile compounds are a group of secondary metabolites that mainly comprises mono and sesquiterpene hydrocarbons and their oxygenated derivatives, aldehydes, esters and alcohols [44]. Plant volatile oils are intended to mediate a plant’s relationship to abiotic agents as salinity, light, drought, temperature, and biotic agents like microbial pests, herbivores, etc. [45,46].

The results showed that the extracts of *A. monosperma* and *L. crithmoides* include different classes of monoterpenes, sesquiterpenes, and hydrocarbons which exhibited the majority of estimated components. The major classes of essential oils (EOs) in *A. monosperma* extract were oxygenated sesquiterpene and oxygenated monoterpenes, while in *L. crithmoides* the major classes of EOs were oxygenated sesquiterpene and hydrocarbons. In this context, it was reported that sesquiterpenes are an essential category of organic constituents produced by plants and are distinctive of Asteraceae. The majority of these constituents are volatiles which play a role in communication and defense against herbivory [47,48]. For example, in *Inula montana* exposed to different abiotic stresses such as drought, large amounts of sesquiterpenes were recorded [49]. Also, the higher concentration of oxygenated monoterpens and comparable concentration of oxygenated sesquiterpene in *A. monosperma* than *L. crithmoides* results in the elevated content of the antioxidant activity of *A. monosperma* compared with *L. crithmoides* [50,51].

γ-eudesmol as bicyclic sesquiterpenoid alkene alcohol was found in all tissues, and it was generally decreasing. It can display moderate activity against human diseases. α-farnesene as another compound can be effective against the bacteria that cause tooth decay [52]. This variety of EOs recorded in *A. monosperma* and *L. crithmoides* matched with other studies which proved the improvement in the SMs was observed in some EO species in response to salinity as in *Mentha pulegium* [53] and rise EOs in *Salvia officinalis* [54]. Thus, the enhancement of oil production in response to salinity can be considered as an adaptation to this stress. In this context, the free volatiles are glycosylated that stored in cell vacuoles and intern rise cellular swelling to reduce the effect of osmotic stress from salinity. Charles and Simon [55] attributed the EOs productivity to higher oil gland density.

Water stress also, causes an improvement in EOs production as in *Salvia officinalis*, *Petroselinum crispum* and *Lippia berlandieri* [56]. This revealed that plants subjected to drought stress progress higher quantities of SMs in their tissues [55]. This could be due to a reduction in the area of the leaves, which leads to increase oil glands and intern an increased amount of oil content under drought stress [57]. In addition, Ben Taarit et al. [58] revealed that stress might raise the number of glands produced previous to leaf appearance by early divisions in leaves epidermal cells. Drought stress causes both cellular and intercellular oxidative stress. Because SMs have strong antioxidant features, they may be associated with a mechanism to combat the harmful effects of reactive-oxygen species (ROS) [41].

## 4. Materials and Methods

### 4.1. Study Site 

The current study was done during August- September 2019 in two sites: site I at New Damietta (latitude 31.42 and longitude 31.82) where *A. monosperma* is growing on sand formation habitats and site II at Abu Qir, Alexandria (latitude 31.31 and longitude 30.06) where *L. crithmoides* is growing on wetlands habitats. Both sites are extended within the central part of the Mediterranean coast of Egypt. The bioclimatic map indicates that the middle region of the Mediterranean coast of Egypt belongs to the sub-desertic warm climate [59]. The mean annual temperature is 20 °C, with annual rainfall varied from 91.6 to 175.2 mm. Mean relative humidity is higher in winter (81%) than in summer (65%).

### 4.2. Soil Sampling and Analysis

A composite soil sample (*n* = 5) at a profile of 30 cm was collected from the growth area of the studied plants. Soil analysis was carried out according to Estefan et al. [60]. The soil moisture was estimated by subtraction of the oven-dry weight of soil from a known fresh-weight and expressed as a percentage of oven-dry weight. Soil texture was detected by the sieves method. Water holding capacity was measured by using the Hilgard’s pan box. The pH and electric conductivity (EC) of the prepared soil extract (1:5 *w/v*) were measured using a multi-parameter meter CONSORT Model 535, handheld. Bicarbonates were estimated through titration against 0.1N HCl with phenolphthalein (ph.ph) indicator. Chlorides content was measured by titration against AgNO_3_ in the presence of potassium dichromate. Sulfates content was spectrophotometrically valued after adding 0.15% calcium chloride dehydrate. Calcium carbonates were measured after precipitation using 0.5 M HCl, then titration against 0.5 M NaOH in the presence of ph indicator. The organic carbon content was detected by titration with standard FeSO_4_ after digestion with chromic and sulphuric acids.

### 4.3. Plant Samples Collection and Analysis

#### 4.3.1. Samples Collection

Healthy samples (*n* = 5) from stem and leaves of *A. monosperma*. and *L. crithmoides* were collected for morphological, anatomical, phytochemicals, antioxidant and essential oils investigations. A part of the plant samples was cleaned by tap water, air-dried and finely grounded for chemical analysis.

#### 4.3.2. Morphological and Anatomical Features

The description of morphological and anatomical features of fresh leaves and stems of *A. monosperma* and *L. crithmoides* was undertaken according to [34,61]. The anatomical assay was performed according to [62] and modified by Peacock and Bradbury [63] as follows: the plant specimen was fixed in a solution of formalin, acetic acid and 70% alcohol (10:5:85, *v*/*v*/*v*). The samples were dehydrated from the fixative by immersing in tertiary butyl alcohol overnight until the dehydration process was completed. Soft paraffin was melted and poured up to two-thirds of vials for the infiltration process and then left to solidify, and returned to the vials and put in an oven (60 °C) until the solvent evaporated. Then soft paraffin wax, moved to hard melted paraffin wax, then placed in the oven (60–62 °C) for two days. Plant samples were put inside the wax, and a refrigerator was used to achieve complete solidification. The block was then fixed in the microtome which was adjusted to a desired thickness (10–15 μm thick). The staining process was carried out by removing paraffin wax from the obtained sections by using xylene (20 min.), followed by a mixture of absolute ethanol and xylene (1:1) for 10 min. The dehydration of the sections was done by transmission through a sequence of 95, 70 and 50% ethanol, respectively. The prepared sections were inundated for one min. in stain (fast green), rinsed with water and transmitted to another stain (safranin for 30 min.). The prepared slides were water-washed and dehydrated using ethanol then sections were cleared in three alterations of xylene and placed in Canada balsam. To remove air bubbles, the slides were put in an oven for a week at 30 °C. Finally, the perfect cross-sections were chosen and examined by an automatic light microscope. Sections were photographed at 40×, 100×, and 400× magnification.

#### 4.3.3. Phytochemicals Analysis 

Total phenols content was measured by the Folin–Ciocalteu (FC) colorimetric method [64]. In concise, a 5 ml FC reagent (10%) and 4 ml of sodium carbonate were added to one ml of ethanolic plant extract. The absorbance was estimated at 765 nm. The concentration of the phenolics was estimated from the calibration curve as g gallic acid equivalents (GAE)/100 g dry wt. Flavonoids content was estimated by the aluminum chloride colorimetric method [64]. Flavonoids were expressed as g catechin (CE)/100 g dry wt. Tannins amount was estimated using the vanillin hydrochloride assay [64]. The standard curve of tannins was set by 0–100 µg of tannic acid (TA). Alkaloids were measured after adding concentrated ammonium hydroxide to the extract until complete precipitation [65].

#### 4.3.4. Antioxidant Activity by Free Radical Scavenging Method

The antioxidant activity of stems and leaves of *A. monosperma*. and *L. crithmoides* was assessed by the scavenging activity of DPPH. The half inhibitory concentration (IC50) of each extract to reduce DPPH was calculated [66].

#### 4.3.5. Essential Oil Analysis

The gas chromatography–mass spectrometry (GC-MS) analysis of the essential oil from the aerial parts of both species was carried out using an instrument with the following specifications: a TRACE GC Ultra Gas Chromatographs (THERMO Scientific Corp., Waltham, USA), coupled with a THERMO mass spectrometer detector (ISQ Single Quadrupole Mass Spectrometer). The GC-MS system was equipped with a TG-5MS column (30 m × 0.25 mm i.d., 0.25 μm film thickness). Analyses were carried out using helium as a carrier gas at a flow rate of 1.0 ml/min and a split ratio of 1:10 using the following temperature program: 60 °C for 1 min; rising at 3.0 °C/min. to 240 °C and held for 1 min. The injector and detector were held at 240 °C. Diluted samples (1:10 hexane, v/v) of 0.2 μL of the mixtures were always injected. Mass spectra were obtained by electron ionization (EI) at 70 eV, using a spectral range of m/z 40–450. Most of the compounds were identified using the analytical method: mass spectra (authentic chemicals, Wiley spectral library collection and NSIT library).

### 4.4. Statistical Analysis

All data were expressed as the mean values ± SE. The statistical significances among soil variables and phytochemical data were tested using the Kruskal–Wallis test (one-way analysis of variance (ANOVA)), followed by Dunn’s method as multiple pairwise comparisons and Bonferroni’s correction. All analyses were performed in the XLSTAT program (version 2016).

## 5. Conclusions

Concerning the ecological field, plant adaption behavior in response to environmental stress (salinity, drought, and low fertility) was evolved in structural and chemical features of plants. *A. monosperma* and *L. crithmoides* from the family Asteraceae showed critical morphological, anatomical, and phytochemical adaptation mechanisms in the Mediterranean coastal habitats of Egypt. As compared with *L. crithmoides*, the high antioxidant capacity of *A. monosperma* may be attributed to their high total phenols, flavonoids, alkaloids and tannins. Three groups of organic essential oils: monoterpenes, sesquiterpenes and hydrocarbons were identified in both species. Local adaptation could be assumed as a guideline or indicator for species’ survival strategy, and this provides critical insights into the reintroduction or recovery conservation planning of the extremely small populations under the harsh conditions.

## Figures and Tables

**Table 1:** Soil properties (mean values ± standard error) supporting the growth of *A. monosperma* (Site I) and *L. crithmoides* (Site II). Different letters in the same row are significantly different at *p* < 0.05.

Scheme	Site I	Site II
Sand (%)	95.20 ± 1.80 ^a^	50.72 ± 0.90 ^b^
Silt (%)	3.50 ± 0.85 ^a^	42.88 ± 1.50 ^b^
Clay (%)	1.20 ± 0.10 ^a^	6.40 ± 0.30 ^b^
Moisture content (MC, %)	3.5 ± 0.90 ^a^	4.70 ± 0.08 ^b^
Water holding capacity (WHC, %)	41.30 ± 1.50 ^a^	62.86 ± 2.30 ^b^
pH	7.81 ± 0.30 ^a^	8.21 ± 0.50 ^a^
Electric conductivity (EC, µS/cm)	6180 ± 112.0 ^a^	7730 ± 67.80 ^b^
HCO_3_ (%)	0.09 ± 0.0 ^a^	0.12 ± 0.0 ^a^
Cl^-^ (%)	0.64 ± 0.02 ^a^	1.10 ± 0.06 ^b^
SO4^-^ (%)	0.65 ± 0.05 ^a^	0.70 ± 0.0 ^a^
CaCO_3_ (%)	11.0 ± 1.00 ^a^	7.0 ± 0.50 ^b^
Organic carbon (OC, %)	0.90 ± 0.01 ^a^	1.40 ± 0.04 ^b^

**Table 2:** Chemical composition of the essential oil (%) from *A. monosperma* and *L. crithmoides*.

No	*A. monosperma* Compounds	RT	MW	MF	Concentration%	*L. crithmoides* Compounds
**Monoterpene hydrocarbons**						
1	*α*-pinene	4.25	136.24	C_10_H_16_	1.56	-
2	(±)-β-pinene	5.45	136.24	C_10_H_16_	6.35	-
3	D-limonene	6.87	136.24	C_10_H_16_	2.09	-
4	*γ*-terpinene	7.81	136.24	C_10_H_16_	1.13	-
5	*p*-cymene	6.79	134.22	C_10_H_14_	1.46	-
6	-	14.59			1.29	*p*-cymene
**Oxygenated monoterpenes**						
7	linalool	9.44	154.25	C_10_H_18_O	2.43	-
8	(−)-*trans*-pinocarveol	11.10	152.24	C_10_H_16_O	3.37	-
9	pinocarvone	12.01	150.22	C_10_H_14_O	2.59	-
10	4-terpineol	12.69	154.25	C_10_H_18_O	2.35	-
11	(−)-myrtenol	13.45	152.23	C_10_H_16_O	3.72	
12	(−)-citronellol	14.66	156.27	C_10_H_20_O	3.01	-
13	-	14.55			1.01	(−)-citronellol
14	-	15.44	148	C_10_H_12_O	1.00	benzaldehyde, 4-(1-methylethyl)-
15	cuminic aldehyde	15.50	148.2	C_10_H_12_O	2.19	-
16	bornyl acetate	16.84	196.29	C_12_H_20_O_2_	1.79	-
17	-	20.88	196.29	C_12_H_20_O_2_	1.67	geranyl acetate
**Oxygenated diterpene**						
18	-	48.83	308.51	C_20_H_34_O_2_	1.24	*cis*-abienol
**Non-oxygenated sesquiterpenes**						
19	-	20.61	204.36	C_15_H_24_	1.51	α-gurjunene
20	*α*-curcumene	22.19	202.34	C_15_H_22_	5.72	-
21	-	24.99			7.49	*α*-curcumene
22	-	25.13	204.36	C_15_H_24_	1.22	*α*-selinene
23		22.17	204.36	C_15_H_24_	1.30	*beta*-caryophyllene
24	-	24.76	204.36	C_15_H_24_	1.46	germacrene D
**Oxygenated sesquiterpenes**						
**25**	-	27.93	220.36	C_15_H_24_O	1.02	ledene oxide-(ii)
26	(−)-caryophyllene oxide	28.02	220.36	C_15_H_24_O	1.35	-
27	-	28.89			2.49	(−)-caryophyllene oxide
28	isoaromadendrene epoxide	28.25	220.36	C_15_H_24_O	1.29	-
29	citronellyl iso-valerate	28.79	240.39	C_15_H_28_O_2_	2.76	-
30	-	28.67			4.09	citronellyl *iso*-valerate
31	(−)-spathulenol	28.99	220.36	C_15_H_24_O	2.21	-
32	-	28.79			2.83	(−)-spathulenol
33	viridiflorol	29.57	222.37	C_15_H_26_O	5.62	-
34	-	29.45			8.15	viridiflorol
35	ledol	29.86	222.37	C_15_H_26_O	1.18	-
36	-	30.07	222.37	C_15_H_26_O	5.66	cubenol
37	widdrol	30.26	222.37	C_15_H_26_O	7.57	-
38	neoclovenoxid-alcohol	30.44	220.36	C_15_H_24_O	1.20	-
39	guaiol	30.92	222.37	C_15_H_26_O	2.40	-
40	-	30.80			1.31	guaiol
41		31.35	222.37	C_15_H_26_O	4.63	tau.-cadinol
42	*γ*-eudesmol or eudesm-4-en-11-ol	32.07	222.37	C_15_H_26_O	14.66	-
43	-	31.88			8.26	γ-eudesmol or eudesm-4-en-11-ol
44	-	32.03	218.34	C_15_H_22_O	1.09	*cis*-nuciferol
45	-	32.32	216.32	C_15_H_20_O	2.44	ar-turmerone
46	-	32.63	224.39	C_15_H_28_O	2.87	6,7-dihydro-2-cis-farnesol
47	-	33.15	220.36	C_15_H_24_O	0.99	aromadendrene oxide-(1)
48	hexahydrofarnesyl acetone	38.34	268.49	C_18_H_36_O	2.39	-
49	-	38.32			2.00	hexahydrofarnesyl acetone
50	-	46.40	222.37	C_15_H_26_O	3.66	(7a-isopropenyl-4,5-dimethyloctahydroinden-4-yl) methanol
**Hydrocarbons**						
51	(1*R*)-(+)-nopinone or .beta-pinone	11.19	138.21	C_9_H_14_O	1.31	-
52	-	15.31	196.29	C_12_H_20_O_2_	2.20	linalyl acetate
53	2,4-pentadiynylbenzene	17.38	140.19	C_11_H_8_	2.37	-
54	naphthalene, 2-ethenyl	26.27	154.21	C_12_H_10_	5.24	-
55	-	26.30			8.83	naphthalene, 2-ethenyl-
56	1,1′-biphenyl	26.98	154.21	C_12_H_10_	6.59	-
57	-	26.49			11.77	1,1′-biphenyl
58	oleamide	54.57	281.48	C_18_H_35_NO	2.10	-
59	-	39.16	278.35	C_16_H_22_O_4_	2.13	diisobutyl phthalate
60		46.40	191	C_10_H_9_NOS	4.39	2-(2-cyanoethylsulfanyl)benzaldehyde
					**Ʃ = 100.0**	

RT: retention time, MW: molecular weight, MF: molecular formula.

## Supplementary Materials

The following are available online at https://www.mdpi.com/2223-7747/10/3/481/s1, Figure S1: Chromatogram and structures of the main components of the essential oils of *A. monosperma*, Figure S2: Chromatogram and structures of the main components of the essential oils of *L. crithmoides*.
