# Supplementary material for: Structural and Chemical Adaptations of Artemisia monosperma Delile and Limbarda crithmoides (L.) Dumort. in Response to Arid Coastal Environments along the Mediterranean Coast of Egypt
Source: Plants (Basel). 2021 Mar 4;10(3):481. doi: 10.3390/plants10030481 (PMC7999453; doi:10.3390/plants10030481)
Supplement: Supplementary file 1 [file plants-10-00481-s001.pdf]

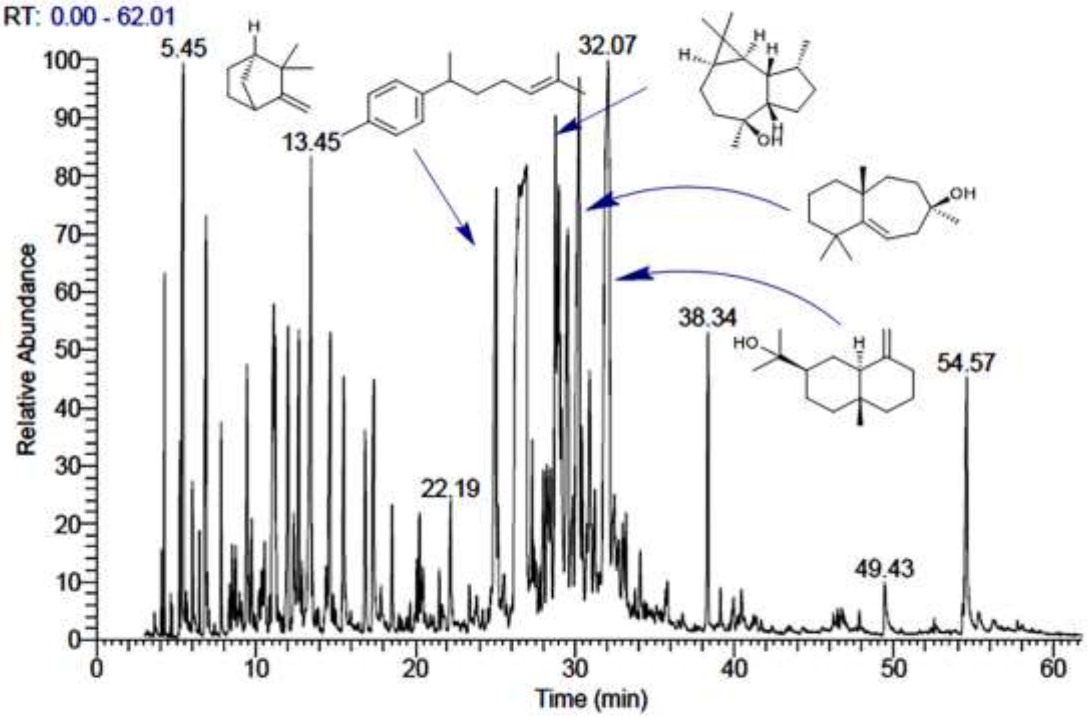

Figure S1. Chromatogram and structures of the main components of the essential oils of *A. monosperma*.

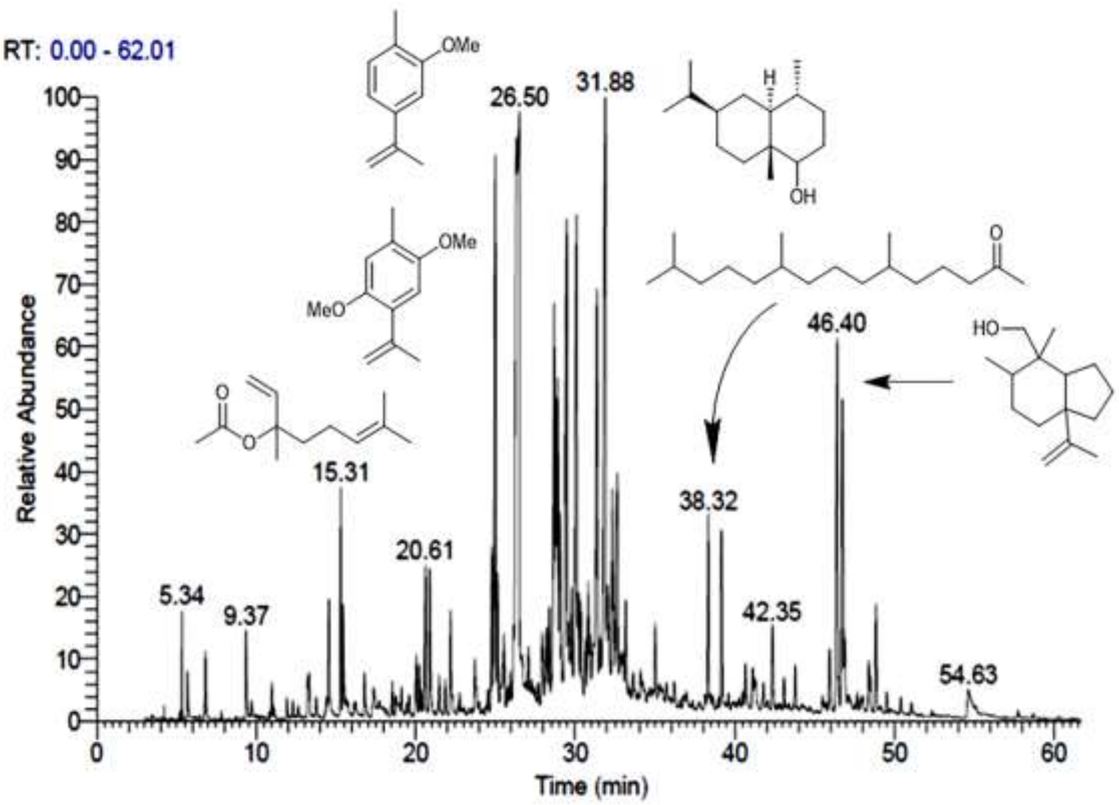

Figure S2. Chromatogram and structures of the main components of the essential oils of *L. crithmoides*.
